# Effects of AI Assistance Timing on Pharmacists’ Trust in Automated Pill Recognition Technology: Within-Participants Experimental Study

**DOI:** 10.2196/92822

**Published:** 2026-09-11

**Authors:** Jin Yong Kim, Brigid Rowell, Megan Whitaker, Qiyuan Chen, Raed Al Kontar, Corey Lester, Xi Jessie Yang

**Affiliations:** 1 Industrial and Operations Engineering, University of Michigan, 1640 IOE, 1205 Beal Ave, Ann Arbor, MI, 48109-1382, United States, 1 7347630541; 2 College of Pharmacy, University of Michigan, Ann Arbor, MI, United States

**Keywords:** artificial intelligence, human-computer interaction, medication verification, medication errors, trust in automation

## Abstract

**Background:**

Image-based pill verification systems demonstrate high model accuracy. However, their effectiveness in pharmacy practice depends on how pharmacists interact with the AI output. The timing of AI advice is one design factor that influences these interactions, yet its impact on pharmacists’ moment-to-moment trust dynamics during medication verification requires further investigation.

**Objective:**

This study aims to investigate how the timing and conditionality of AI assistance shape pharmacists’ trust dynamics during medication verification.

**Methods:**

Between April and December 2024, 50 licensed pharmacists completed a browser-based simulated medication dispensing task with 2 AI types: ex-ante advice (AI advice given concurrently with clinical information) and ex-post advice (AI advice given after an initial diagnosis). Ex-post advice was further divided into the involved ex-post and the not-involved ex-post conditions. The experiment used a within-participants design with varying AI types and AI recognition patterns (right fill-correct recognition, right fill-incorrect recognition, wrong fill-correct recognition, and wrong fill-incorrect recognition). The primary outcomes were trust adjustment magnitude and trust adjustment, which were analyzed using mixed-effects linear regression models.

**Results:**

Trust adjustment magnitude differed significantly across AI assistance conditions. The involved ex-post condition led to the highest magnitude of trust adjustment, followed by ex-ante advice (mean difference 9.65, 95% CI 8.55-10.75; *P*<*.*001), with the not-involved ex-post condition showing the lowest magnitude (mean difference 3.33, 95% CI 2.63-4.03; *P*<*.*001). Analysis by each recognition pattern revealed significant differences in trust adjustment when the right drugs were incorrectly rejected. In this pattern, the involved ex-post condition led to larger trust decrements than both ex-ante advice (mean difference −2.13, 95% CI −3.83 to −0.44; *P*=.008) and not-involved ex-post conditions (mean difference −7.32, 95% CI −13.69 to −.95; *P*=.009). A marginal difference was observed between ex-ante advice and not-involved ex-post conditions (mean difference −5.19, 95% CI −11.55 to 1.17; *P*=.051). No significant differences were observed for other recognition patterns.

**Conclusions:**

Both the timing and conditionality of AI assistance influenced pharmacists’ trust dynamics. Disagreement-based AI interventions (involved ex-post) that incorrectly challenged pharmacists led to a substantial trust decrement, whereas the not-involved AI intervention resulted in more stable trust fluctuations. These findings highlight the importance of designing AI systems that align intervention strategies with user expertise and task demands to foster appropriate trust in safety-critical workflows.

## Introduction

### Background

Pharmacists play a critical role in ensuring that patients receive the correct medication, particularly during medication verification, in which the medication filled inside the bottle must match the prescription label [1]. Errors at this stage, such as dispensing the wrong drug, strength, or formulation, are a major source of preventable adverse drug events, with error rates that can exceed 1% in both community and hospital settings [2-4]. These errors adversely impact patient safety and trigger emergency room visits and hospitalizations [5]. Contributing factors include high workload, fatigue, technology limitations, lack of trust in automation, and the high cognitive demands placed on pharmacists who must manage multiple tasks simultaneously [6-9]. Because medication verification is both labor-intensive and cognitively demanding, tools that reduce dispensing errors have the potential to substantially improve patient outcomes and decrease health care costs [10].

To mitigate these risks, image-based pill verification systems have been introduced [11-15]. Advances in deep learning have further improved the performance of these systems, with recent models achieving accuracies greater than 98% [16]. However, the successful integration of AI systems into pharmacy workflows depends not only on model accuracy but also on how humans perceive, trust, interpret, and act on AI output [8,17,18]. Prior human factors and human-computer interaction (HCI) research highlights two design dimensions that strongly influence human-AI interaction: (1) the amount and type of model information presented and (2) the timing of AI assistance [19,20]. These dimensions shape cognitive load, situational awareness, and the development of trust in autonomous and AI systems, defined as the belief that an autonomous or AI agent will support an individual’s goals under conditions of uncertainty [21].

The amount and type of model information can play a key role in promoting clinician understanding of clinical decision support systems (CDSSs). To overcome the barrier of the “black-box” nature of many of these CDSSs, in which model outputs provide little insight into how predictions are generated, research has emphasized the importance of enhancing system transparency through explanatory feedback and uncertainty visualizations [9,22-26]. For instance, Kompa et al [26] investigated how different levels of explanation in a CDSS supporting balance disorder diagnosis influenced health care professionals’ trust and reliance. Participants used either a selective CDSS that presented examination results alone or a comprehensive CDSS that additionally provided explanatory context. Their findings showed that explanations positively affected trust, whereas limited explanations led users to question the system’s reliability [26]. Kim et al [9] examined how presenting model uncertainty information in a medication verification AI affected pharmacists’ trust. Their pill characteristic plots conveyed match-mismatch information based on visual attributes such as imprint, color, shape, and score, and the uncertainty plot illustrated the predicted probability of each set of predictions summarized in a histogram [11]. By manipulating the presence of uncertainty visualization as a between-participants factor, they found that communicating uncertainty improved pharmacists’ end trust, suggesting that transparency fosters more calibrated trust and active engagement with AI systems [9].

Another critical design factor influencing human-AI interaction is the timing of AI advice [20]. In clinical decision-making, AI systems may present recommendations before the human makes the initial decision (ex-ante AI advice) or after the human forms an initial judgment (ex-post AI advice) [19,27]. Ex-ante systems provide continuous feedback throughout the task [28,29], supporting real-time integration of AI insights, but increasing the risk of cognitive overload or anchoring when AI outputs are incorrect [30-32]. In contrast, ex-post systems require the human decision maker to commit to an initial decision before receiving AI advice [33-36]. While this approach can preserve human decision autonomy and reduce the anchoring effect, it may exacerbate confirmation bias [37-39], contribute to algorithm aversion [35,40,41], and introduce workflow interruptions when AI intervenes unnecessarily [42].

Empirical findings on the optimal timing of AI assistance remain mixed. Yin et al [27] investigated how the timing of AI advice affects doctors’ diagnostic decision-making. In their experiment, they recruited and asked physicians to diagnose clinical cases. The authors compared ex-post advice with ex-ante advice. They found that physicians engaged more deeply with ex-post advice, resulting in higher diagnostic accuracy (task performance) compared with ex-ante advice. Conversely, Fogliato et al [43] explored a human-AI workflow sequence with veterinary radiologists, who reviewed X-ray images and made diagnostic judgments under two conditions: (1) a 1-step workflow (ex-ante advice), where AI inferences were shown before radiologists’ diagnosis and (2) a 2-step workflow (ex-post advice), where radiologists made an initial diagnosis first, then saw the AI’s recommendations. The authors reported that radiologists’ diagnostic performance was better in the ex-ante advice system compared to the ex-post advice system. With the ex-post advice help, radiologists rarely revised their initial judgments and rated the AI as less useful [43].

### Properties of Trust Dynamics

Trust in autonomous and AI systems has been extensively studied across domains, including health care [9,17,21]. Trust is a critical determinant of how people rely on autonomous systems and is strongly influenced by system performance [44-46].

Most existing literature has adopted a snapshot approach to trust measurement, typically assessing trust through posttask or end-of-experiment questionnaires. While informative, this approach overlooks that trust is a dynamic variable and evolves over the course of repeated, moment-to-moment interactions with autonomous systems [45,47,48].

Yang et al [45] identified and summarized 3 key trust dynamics properties. First, continuity suggests that trust at a given moment (*i*) is strongly associated with trust at the immediately preceding moment (*i −* 1). Trust tends to increase following automation successes and decrease following failures [9,46]. Second, negativity bias highlights the asymmetric impact of autonomy performance, wherein trust is difficult to build but can be lost quickly. Prior studies have shown that negative experiences with automation failures exert a stronger influence on trust than positive experiences [8,49-51]. Third, stabilization refers to the tendency for trust to stabilize over repeated interactions with the same system, as users gain familiarity and form more stable expectations [52].

### Present Study

Building on these findings, the present study investigates how the timing of AI assistance shapes pharmacists’ trust dynamics during medication verification. We examine 2 modes of AI engagement: ex-post advice (AI advice given after an initial diagnosis) and ex-ante advice (AI advice given concurrently with clinical information). The ex-ante advice continuously displays predictive information throughout the task, and the ex-post advice intervenes only when its prediction conflicts with the pharmacist’s initial decision. Ex-ante advice may support continuous decision-making but risks cognitive overload and anchoring, whereas ex-post advice may reduce unnecessary attentional demands but risks disruptive false alarms [30,32-34,36].

By comparing these timing strategies, we aim to identify how different modes of AI engagement influence trust adjustment in a safety-critical and expert-driven medication verification task.

In addition, prior empirical validations of trust dynamics properties (continuity, negativity bias, and stabilization) have relied primarily on simplified tasks and nonexpert participant populations [8,45,48]. By examining licensed pharmacists engaged in realistic medication verification tasks, the present study extends prior work and strengthens the ecological validity of trust dynamics properties in a safety-critical health care context.

## Methods

### Ethical Considerations

This study was exempt from institutional review board oversight by the University of Michigan (HUM#00241223). All participants provided electronic informed consent prior to the experiment, and data were collected anonymously. Participants received US $100 upon completing the study.

### Participants

Pharmacists were recruited via email through the Minnesota Pharmacy Practice-Based Research Network, Pharmacy Practice Enhancement and Active Research Link in Wisconsin, and the University of Michigan College of Pharmacy Pharmacist Preceptor Network. Participants were eligible for the study if they were licensed in the United States, were at least 18 years old, and had access to a laptop or desktop computer with a webcam. Pharmacists were excluded if they needed assistive technology to operate a computer, wore eyeglasses with more than 1 power, had uncorrected cataracts, intraocular implants, glaucoma, or permanently dilated pupils, and/or had eye movement or alignment abnormalities (eg, lazy eye, strabismus, nystagmus). Fifty licensed US pharmacists participated in the study between April and December 2024.

### AI Model

The AI model used in this study is a Bayesian neural network (BNN) designed to predict the National Drug Code (NDC) for dispensed pills while estimating the prediction uncertainty [11,16]. This was achieved by applying random dropout to a ResNet-34 convolutional neural network architecture [53,54]. Instead of outputting a single probability distribution for each class, the model generated 50 probability vectors for each image. The final NDC prediction was determined by averaging those vectors and selecting the class with the highest mean probability.

The model was trained on a dataset of 432,974 images representing 352 distinct NDC classes from a mail-order pharmacy in the United States, which uses a robotic system to count pills, fill and label vials, capture images of the contents, and seal the vials [55]. The dataset contains 1 year of robot-captured images of solid oral medication (ie, tablets and capsules) inside prescription vials, with each image associated with its corresponding NDC and annotated with attributes such as color, shape, size, manufacturer, tablet scoring, and imprint. The number of available images for each NDC varied from 3 to 12105, with a median of 540 (IQR 257-1291).

The medications in the dataset were categorized into 12 distinct colors: white (42.1%), yellow (12.3%), pink (9.1%), orange (7.1%), multicolor (5.9%), green (5.2%), red (5.2%), blue (4.8%), brown (3.8%), purple (3.1%), turquoise (0.7%), and gray (0.7%). Additionally, seven unique shapes were categorized: round (49.6%), oval (33.4%), capsule (16.2%), hexagon-6-sided (0.4%), triangle (0.3%), trapezoid (0.1%), and pentagon-5-sided (0.04%).

### Experimental Apparatus

#### Overview

The study utilized Labvanced, a browser-based experimental platform, to conduct simulated medication dispensing tasks. Participants accessed the testbed through a web browser on their personal computers, which enabled the software to collect eye-tracking data through the webcam while also recording response decisions and reaction times for each task.

#### Experimental Task

In this experiment, participants performed a simulated medication verification task supported by varying types of imperfect automated pill recognition systems [9,12,13]. The user interface displayed a reference image of prescription medication, the prescription details, and an image of the filled medication. In the 2 AI-assistance conditions, the AI-generated decision support was also presented. The objective was to determine whether the filled medication matched the prescription (ie, reference image). If the 2 images matched, the correct response was to click “accept”; if they differed, the appropriate response was to click “reject.”

The experimental stimuli, including the reference NDC and corresponding filled medication images, were carefully selected from the dataset of 432,974 images. The selection prioritized a broad representation of medication colors and shapes while excluding blurry images to ensure clarity. To reduce potential learning effects, each reference NDC was presented no more than twice during the experiment. To ensure sufficient exposure to both correct and incorrect AI predictions, we constructed a 300-image experimental dataset (representing 154 distinct NDCs) by selectively sampling from the larger dataset to increase the proportion of naturally occurring incorrect predictions, resulting in an overall AI accuracy of approximately 79%. NDCs could occur no more than twice to prevent bias. AI outputs were not artificially modified, and all predictions reflect the model’s original performance. Because this sampling approach preserved the model’s inherent error patterns, prediction errors were not uniformly distributed across medication characteristics (see Table 1).

**Table 1.** Distribution of medication characteristics for the experimental dataset (n=300).

Characteristics	Filled, n (%)	Reference, n (%)
Color		
White	154 (51)	160 (53)
Yellow	42 (14)	34 (11)
Pink	24 (8)	22 (7)
Orange	21 (7)	25 (8)
Multicolor	4 (1)	4 (1)
Green	14 (5)	14 (5)
Red	7 (2)	7 (2)
Blue	12 (4)	12 (4)
Brown	14 (5)	14 (5)
Purple	4 (1)	4 (1)
Turquoise	4 (1)	4 (1)
Shape		
Round	181 (60)	187 (62)
Oval	80 (27)	72 (24)
Capsule	35 (12)	37 (12)
Hexagon (6-sided)	2 (1)	2 (1)
Triangle	2 (1)	2 (1)

To characterize the nature of AI errors, the visual similarity between the reference medication and the AI-predicted medication was examined. Most incorrect predictions (93%) occurred between medications sharing the same color and shape (ie, visually similar medications with different NDCs). A small proportion involved a shape mismatch (5%) or a color mismatch (2%), and none involved mismatches in both color and shape.

**Figure 1.**
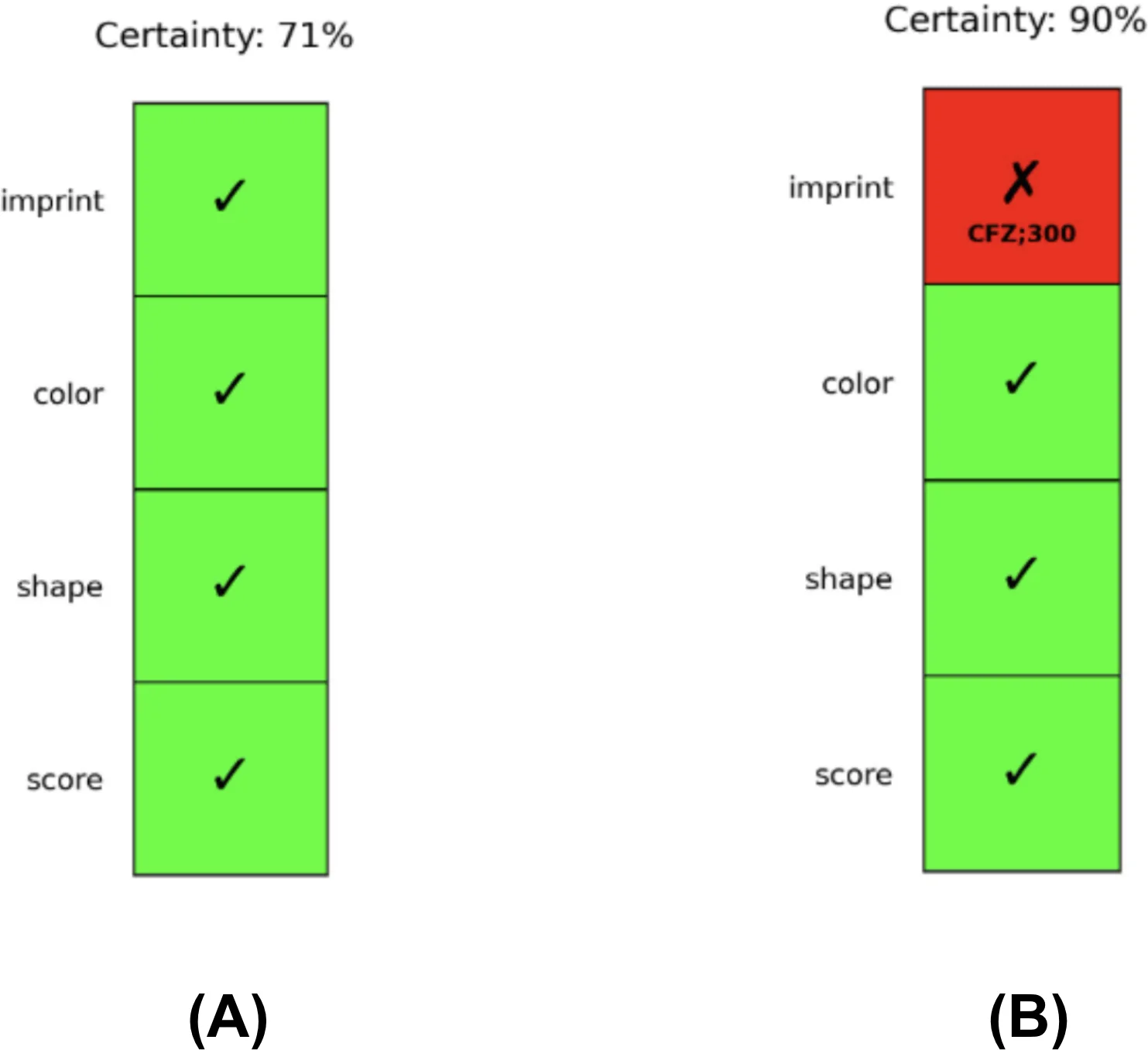
Checkbox plots. (A) Checkbox plot indicating a match and (B) checkbox plot indicating a mismatch. Matching characteristics were represented using green checkboxes with checkmarks. Mismatches were represented using red checkboxes marked with an “X,” along with a textual description of the AI-predicted characteristic.

Two types of AI assistance, both powered by the same underlying BNN model, were integrated into the interface. The AI model communicated its confidence (or uncertainty) and feature matches through a checkbox plot indicating 4 key pill characteristics: imprint, color, shape, and score (Figure 1A). Matching characteristics were represented using green checkboxes with checkmarks. Mismatches were represented using red checkboxes marked with an “X,” along with a textual description of the AI-predicted characteristic (eg, the predicted imprint shown in Figure 1B). The AI’s confidence was represented by the predicted probability of the highest-ranked NDC class.

**Figure 2.**
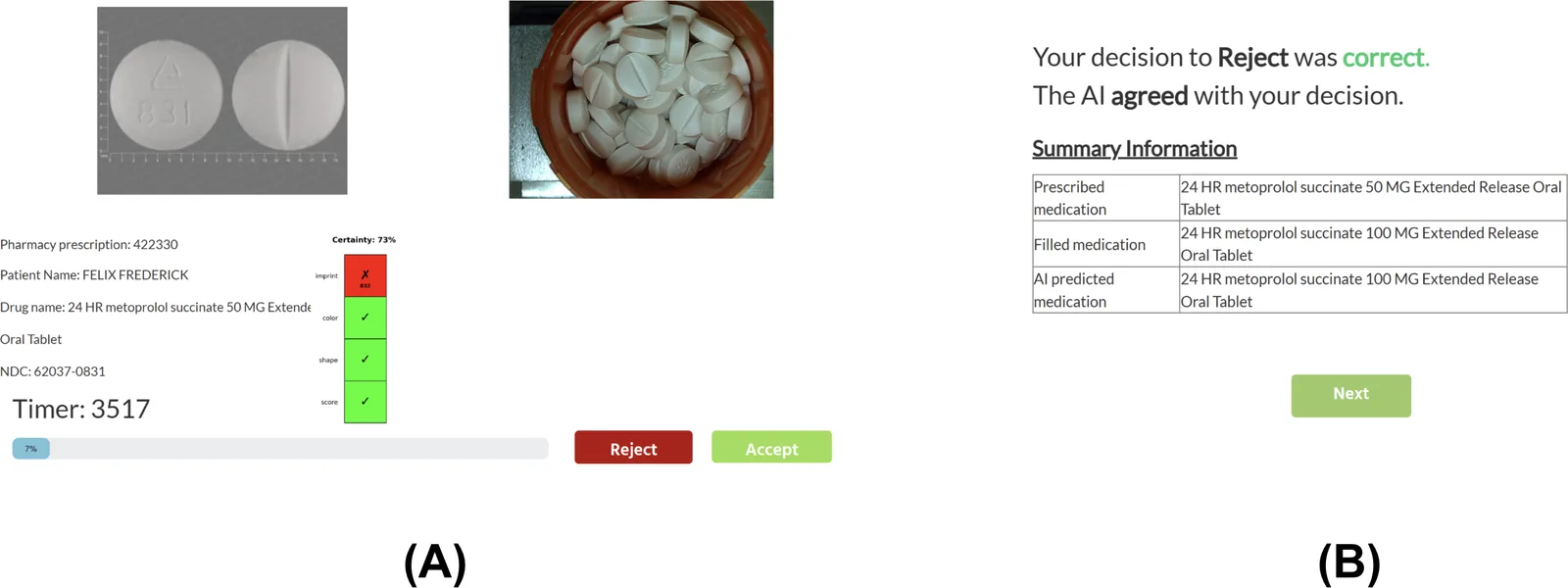
The ex-ante advice condition displayed the checkbox plot before the participant made a decision. (A) Ex-ante advice selection and (B) ex-ante advice feedback. Feedback was displayed after the initial decision.

**Figure 3.**
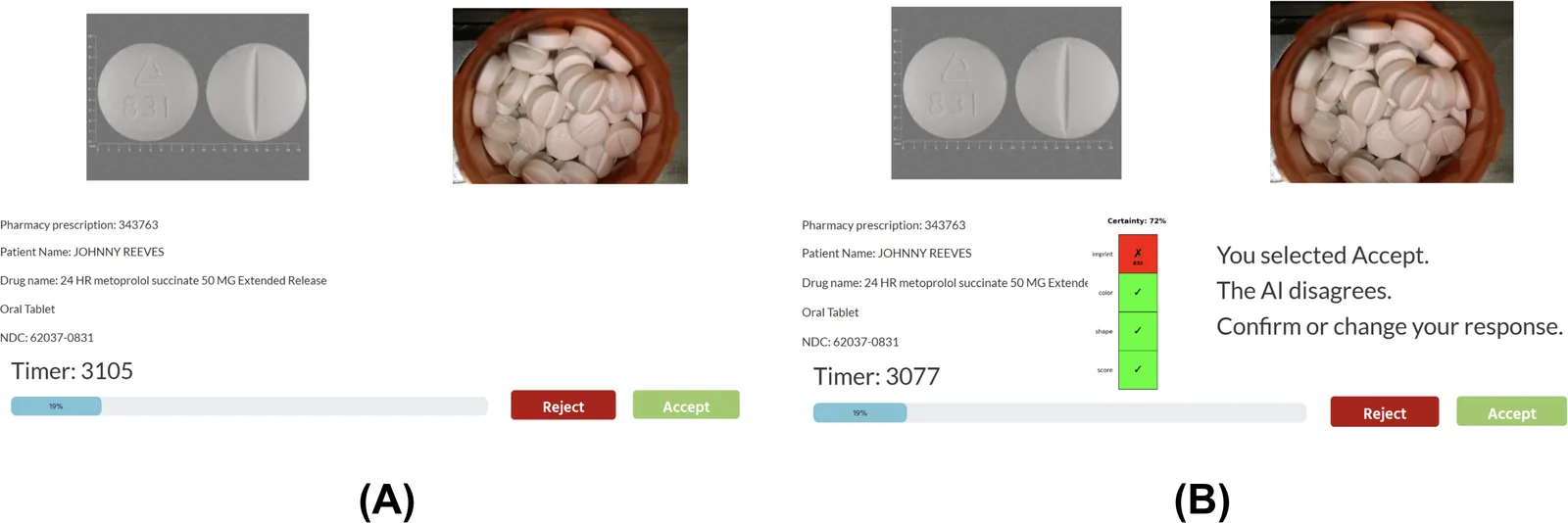
The ex-post advice condition served as a “safeguard,” appearing only when the participant’s response contradicted the AI’s prediction. (A) Ex-post advice initial selection and (B) ex-post advice intervention.

The ex-ante advice condition continuously displayed the checkbox plot before the participant made a decision (Figure 2A). Feedback was displayed after the participant made the decision, indicating the accuracy of the participant’s selection and the AI’s prediction (Figure 2B). On the other hand, the ex-post advice condition served as a “safeguard,” appearing only when the participant’s response contradicted the AI’s prediction. For example, if the participant initially selected “accept” (Figure 3A) when the AI predicted a mismatch, the AI intervened and prompted a reverification by displaying a message, such as: “You selected Accept. The AI disagrees. Confirm or change your response” (Figure 3B). The AI assistance was intentionally imperfect, occasionally providing incorrect recommendations. Although the AI model’s accuracy was 98.5%, it was deliberately lowered to 79% to ensure sufficient exposure to both AI’s errors and AI’s correct guidance outputs. This manipulation enabled the systematic observation of pharmacists’ responses to AI successes and failures. The selected reliability level was informed by prior human factors research suggesting that when automation reliability falls below approximately 70%, its costs may outweigh its benefits, highlighting the importance of studying user responses across a range of imperfect, yet still functional, automation performance levels [56].

Participants completed 3 blocks of 100 medication verification trials, with each block corresponding to each AI assistance condition, including a no-assistance control condition. In the AI-assisted blocks, participants received trial-by-trial feedback on indicating whether their decision to accept or reject was correct and whether the AI’s recommendation was accurate (ie, “Your decision to accept was correct. The AI agreed with your decision”). Following each AI-assisted trial, participants rated their trust in the AI using a visual analog scale ranging from 0 to 100, anchored by “Not at all trust” (0) on the far left to “Completely trust” (100) on the far right. Because the no-assistance control condition did not involve AI support, no trust ratings were obtained.

### Experimental Design

The experiment used a within-participants design with varying AI types (ex-ante advice and ex-post advice) and AI recognition patterns (right fill-correct recognition, right fill-incorrect recognition, wrong fill-correct recognition, and wrong fill-incorrect recognition). Within the ex-post advice condition, AI assistance was further classified as the involved ex-post condition (ex-post-AI disagreed with the human decision) and the not-involved ex-post condition (ex-post-AI agreed with the human decision). The agreement conditions depended on participants’ responses and therefore could not be predetermined. Block order was counterbalanced across participants using a Latin square design. Each AI type block consisted of 100 medication verification trials randomly selected by the experimental platform from the larger trial pool, including 60 right fill-correct recognition, 16 right fill-incorrect recognition, 19 wrong fill-correct recognition, and 5 wrong fill-incorrect recognition trials.

### Measures

After each trial *i*, participants report their *trust*(*i*) in the AI. We calculate a trust adjustment as:

*Trust adjustment*(*i*) = *Trust*(*i*) *− Trust*(*i −* 1), where *i* = 2, 3, *...*, 100

Since the moment-to-moment trust is reported after each trial, 99 trust adjustments are obtained from each participant.

Participant demographics, trust propensity, and eye-tracking data were also collected. Trust propensity was evaluated as a potential covariate during model development, but was not a significant predictor and did not alter the results; therefore, it was excluded from the final models. Because the present study focuses on within-participant trust adjustment, demographic and eye-tracking analyses are beyond the scope of this paper and are reported in a companion manuscript.

### Experimental Procedure

Before the experiment, participants met with a member of the research team via Zoom (Zoom Communications Inc.) to verify that technical and environmental requirements were met, including a functioning webcam, adequate lighting, and a quiet workspace. Once confirmed, participants received a secure link and password to access the Labvanced software. An instructional video then introduced the mock verification process using the study interface, explaining that the objective was to determine whether an image of a filled medication vial matched the prescribed reference image.

The video also outlined the 2 AI assistance features.

Prior to starting the verification tasks, participants completed a demographic questionnaire, the Occupational Fatigue Exhaustion/Recovery Scale (OFER), and the trust propensity survey, followed by calibration procedures for the eye-tracking software [57,58].

During each trial, a “reference image” (the prescribed medication) appeared on the left, and a “filled vial image” (the dispensed medication) appeared on the right, along with relevant prescription details (Figure 2A). Participants decided whether to accept or reject the filled medication based on this comparison. Each participant completed 100 mock verification trials under the ex-ante advice condition (Figure 2A), 100 trials under the ex-post advice condition (Figure 3B), and 100 trials without AI assistance, in a counterbalanced order. The 6 block orders were assigned approximately evenly across 50 participants (4 sequences: n=8 each; 2 sequences: n=9 each). After each block of 100 trials, participants completed postcondition surveys, including the trust survey on a 5-point Likert scale, the system usability scale, the NASA Task Load Index (NASA-TLX) survey, and nonmandatory free-response feedback questions [59-61].

### Analysis

We analyzed trust adjustment magnitude and trust adjustment using mixed-effects linear regression models. The models included categorical predictors and random intercepts to account for repeated measures within participants. Predictors included AI type and AI recognition pattern, forming a 3 × 4 design. We first examined differences between AI types (ex-ante advice and ex-post advice) and then further decomposed the ex-post advice into the involved ex-post condition and the not-involved ex-post condition for a 3-level comparison between interaction conditions. This decomposition was prespecified to examine the trust-dynamics associated with disagreement-triggered versus agreement-based AI interactions. Because assignment to the involved and not-involved conditions depends on pharmacists’ initial decisions, these comparisons were response-contingent and are treated as exploratory analyses rather than confirmatory tests.

AI recognition patterns consisted of 4 levels: right fill-correct recognition, right fill-incorrect recognition, wrong fill-correct recognition, and wrong fill-incorrect recognition. Because the study focused on pharmacists’ trust in AI-assisted decision-making, we restricted the analysis to AI-assisted conditions and excluded data from the no-AI-assistance control block.

We conducted regression 2-tailed *t* tests to compare mean trust outcomes across AI types within each AI recognition pattern. To account for multiple pairwise comparisons, *P* values for post hoc comparisons were adjusted using the Benjamini-Hochberg false discovery rate procedure. We conducted all analyses using mixed-effects linear regression models implemented in the lme4 package, applying the Kenward-Roger method to estimate degrees of freedom [62]. All analyses were conducted in the R statistical software (version 4.2.2) [63].

## Results

### Overview

Observations with trust-adjustment values exceeding ±3 SDs from the mean were excluded prior to analysis to reduce the influence of extreme outliers. To evaluate order effects, block order was included as a fixed-effect covariate. The likelihood ratio test showed that adding block order did not improve model fit for either outcome (trust adjustment magnitude: *χ*^2^_9_=8.88*; P*=.11; trust adjustment: *χ*^2^_9_=6.25; *P*=.28). Therefore, the results from the model excluding block order are presented.

### Comparison Between AI Types

#### Trust Adjustment Magnitude Between Ex-Ante and Ex-Post Advice

AI type (*F*_1_*_,_*_9562.1_=3.45*, η*^2^=0.0004; *P*=.06) marginally affected the magnitude of trust adjustment. Ex-ante advice (estimated marginal mean [EMM] 6.13, 95% CI 4.28-7.97) led to marginally higher trust adjustment magnitude compared to ex-post advice (EMM 5.60, 95% CI 3.76-7.45; Table 2).

**Table 2.** Mean and SD of trust adjustment magnitude and trust change for different AI recognition patterns. AI recognition pattern to confusion matrix representation^a^.

AI type and AI recognition pattern (fill accuracy - (human) AI)	Trust adjustment magnitude	Trust adjustment	Observations, n
Ex-ante advice	5.94 (15.23)		
Right-correct		2.14 (9.03)	2868
Wrong-correct		1.60 (9.90)	1088
Right-incorrect		−15.98 (26.98)	76
Wrong-incorrect		−12.78 (19.85)	800
Ex-post advice	5.37 (15.23)		
Right-correct		1.83 (8.47)	2841
Wrong-correct		1.68 (7.64)	1045
Right-incorrect		−17.84 (29.24)	90
Wrong-incorrect		−9.57 (16.71)	798
Involved Ex-Post	15.44 (26.36)		
Right-(incorrect)-correct		0.07 (13.74)	99
Wrong-(incorrect)-correct		2.75 (7.57)	95
Right-(correct)-incorrect		−18.09 (29.33)	68
Wrong-(correct)-incorrect		−8.96 (16.70)	769
Not-involved Ex-Post	2.60 (8.31)		
Right-(correct)-correct		1.90 (8.21)	2742
Wrong-(correct)-correct		1.57 (7.64)	950
Right-(incorrect)-incorrect		−11.14 (26.06)	22
Wrong-(incorrect)-incorrect		−11.45 (16.97)	29

a AI recognition pattern to confusion matrix representation. 472 right fill and correct recognition: correct rejection; wrong fill and correct recognition: correct detection; 473 right fill and incorrect recognition: false positive; wrong fill and incorrect recognition: false negative.

#### Trust Adjustment Magnitude Between Ex-Ante, Involved Ex-Post, and Not-Involved Ex-Post

When ex-post advice was further separated into involved and not-involved ex-post, the interaction condition (*F*_2,9565.1_=380, *η*^2^=0.07; *P<.*001) significantly affected the magnitude of trust adjustment.

A post hoc comparison between interaction conditions revealed that involved ex-post condition (EMM 15.78, 95% CI 13.62-17.94) led to significantly higher trust adjustment magnitude compared to ex-ante advice (EMM 6.13, 95% CI 4.15-8.11; *t*_9555.51_=21.00*, *mean difference 9.65, 95% CI 8.55-10.75, Cohen *d*=0.72; *P<.*001) and not-involved ex-post condition (EMM 2.80, 95% CI 0.80-4.79; *t*_9556.27_=27.55*, *mean difference 12.98, 95% CI 11.85-14.11, Cohen *d*=0.97; *P<.*001). Ex-ante advice led to a higher trust adjustment magnitude compared to not-involved ex-post condition (*t*_9554.52_=11.46*, *mean difference 3.33, 95% CI 2.63-4.03, Cohen *d*=.25; *P<.*001; Table 2).

### Comparison Between AI Types Within Each Recognition Pattern

#### Trust Adjustment Between Ex-Ante and Ex-Post Advice for Each Recognition Pattern

Recognition pattern (*F*_3_*_,_*_9555.9_=811.37*, η*^2^=0.2; *P<.*001) significantly affected trust adjustment; however, AI type did not influence trust adjustment (*F*_1_*_,_*_9548.6_=2.47*, η*^2^=0.0003; *P*=.12). However, a marginal interaction effect was observed (*F*_3_*_,_*_9550.7_=2.52*, η*^2^=0.0008; *P*=.06).

Because of the marginal interaction effect, we compare Ex-Ante and ex-post advice within each recognition pattern. Significant differences were observed when the right drugs were incorrectly rejected. Ex-post advice (EMM *−*17.84, 95% CI *−*19.06 to *−*16.62) showed larger trust decrement compared to ex-ante advice (EMM *−*15.97, 95% CI −17.19 to −14.76; *t*_9556.76_=*−*2.67*, *mean difference *−*1.87, 95% CI −3.24 to −0.49, Cohen *d*=*−*0.13; *P*=.008). For other recognition patterns, no significant differences were observed.

#### Trust Adjustment Between Ex-Ante, Involved Ex-Post, and Not-Involved Ex-Post for Each Recognition Pattern

Both interaction condition (*F*_2_*_,_*_9558.7_=3.89*, η*^2^=0.0008; *P*=.03) and recognition pattern (*F*_3_*_,_*_9553.7_=484.11*, η*^2^=0.13; *P<.*001) significantly affected trust adjustment. In addition, a significant interaction effect was observed (*F*_6_*_,_*_9570.8_=2.12*, η*^2^=.0013; *P*=.048).

Because of the significant interaction effect, we compared interaction conditions within each recognition pattern. Significant differences were observed when the right drugs were incorrectly rejected. Involved ex-post condition (EMM −18.11, 95% CI −19.34 to −16.87) showed larger trust decrement compared to ex-ante advice (EMM −15.97, 95% CI −17.19 to −14.76; *t*_9553.27_=*−*3.02*, *mean difference −2.13, 95% CI −3.83 to −0.44, Cohen *d*=−0.15; *P*=.008) and not-involved ex-post condition (EMM −10.79, 95% CI −15.96 to −5.61; *t*_9577.26_=*−*2.75*, *mean difference −7.32, 95% CI −13.69 to −0.95, Cohen *d*=−0.52; *P*=.009). A marginal difference was observed between ex-ante advice and not-involved ex-post condition (*t*_9569.61_=*−*1.94, mean difference −5.19, 95% CI −11.55 to 1.17, Cohen *d*=−0.37; *P*=.052). For other recognition patterns, no significant differences were observed.

### Evaluating Trust Properties: Continuity, Negativity Bias, and Stabilization

To explore the key characteristics of trust dynamics, the recognition patterns were grouped into 2 categories: correct AI predictions (right drug correctly approved and *wrong* drug correctly rejected) and incorrect AI predictions (right drug *incorrectly* rejected and *wrong* drug *incorrectly* accepted).

#### Continuity

**Figure 4.**
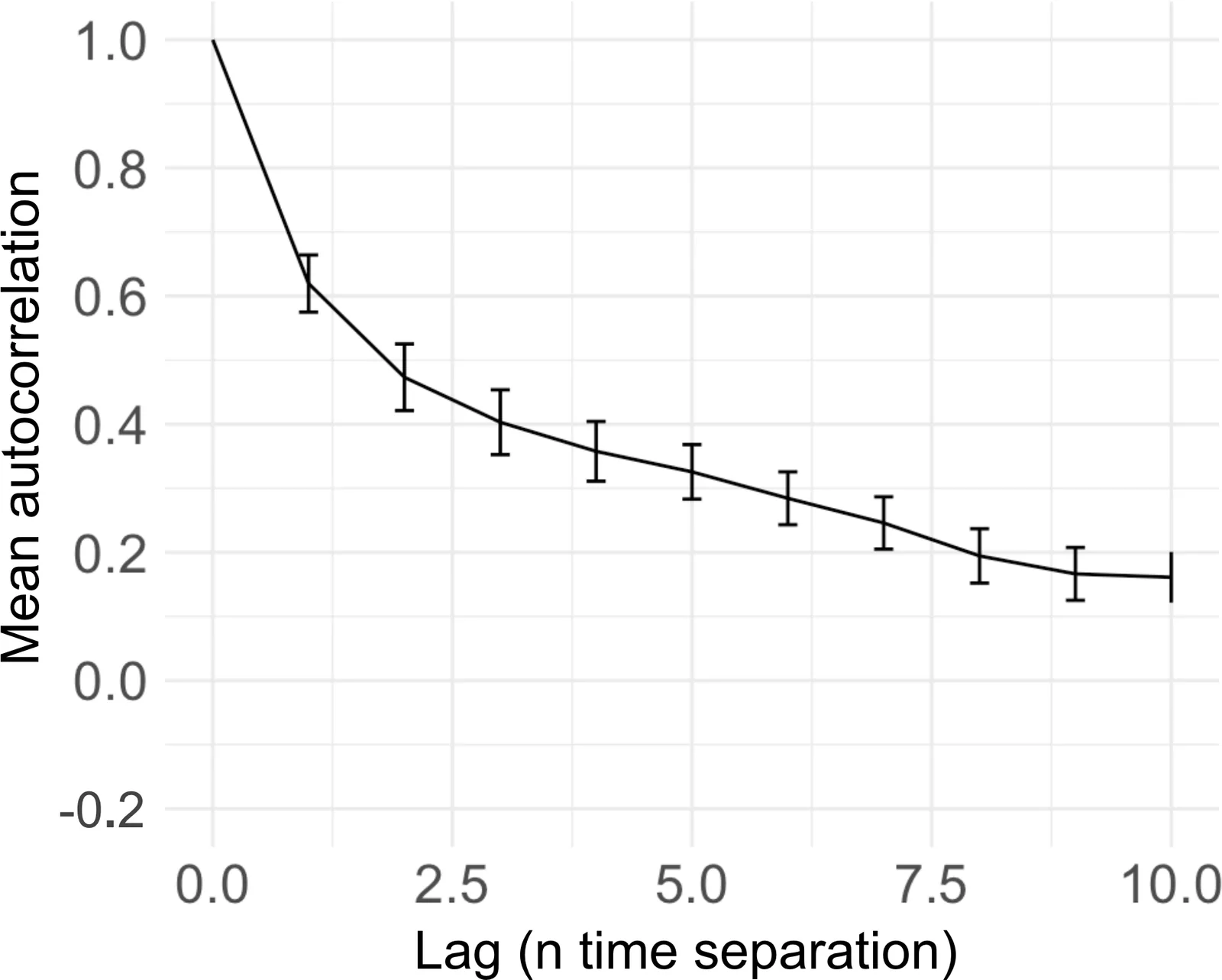
Autocorrelation of trust as a function of time separation. The error bars represent ±2 SEs.

Comparing instances of correct versus incorrect AI predictions revealed that successful AI predictions generally increased user trust, while failures led to decreases. In addition, trust levels at a given moment (*t*) were strongly correlated with trust from the preceding moment (*t −* 1). To assess how current trust ratings relate to past ones, we calculated the autocorrelation of trust ratings. As shown in Figure 4, trust at time *t* closely aligns with trust at time *t −* 1. However, as the time interval between ratings grew longer, the average autocorrelation declined, indicating that trust correlation weakens over time (Figure 4).

#### Negativity Bias

To test for negativity bias, we compared the magnitude of trust increases following correct predictions with the magnitude of trust decreases following incorrect ones. The results indicated a significant difference between the two: correct AI predictions (mean 2.98, SD 7.33) produced smaller changes than incorrect ones (mean 15.80, SD 23.60) (*t*_4726_=30.59*, *mean difference −12.7, 95% CI −13.6 to −11.9, standardized effect size=−1.15; *P<.*001).

#### Stabilization

To examine the stabilization property, linear mixed models were used to analyze trust adjustments over time (sequential success [or failure] occurrence).

The findings revealed that the magnitude of trust adjustments decreased significantly across occurrences for both correct predictions (β=*−*.037*,* 95% CI *−*0.055* to −*0.019, *t*_4021.10_=*−*4.10; *P<.*001) and incorrect predictions (β=*−*.402*,* 95% CI −0.659 to −0.155, *t*_827.46_=*−*3.20*; P*=.001).

## Discussion

### Principal Findings

This study investigated how the timing of AI assistance shaped pharmacists’ trust during AI-assisted medication dispensing. We implemented two AI advice timing conditions. In the ex-ante advice condition, the AI presented recognition predictions before pharmacists’ initial decision. In the ex-post advice condition, the AI provided selective, postdecision intervention. The ex-post advice was further separated into the involved ex-post condition, which intervened when disagreed with the pharmacist, and the not-involved ex-post condition, which withheld intervention when in agreement.

Overall, differences in trust adjustment magnitude across AI types were modest, with ex-ante advice eliciting slightly higher trust adjustment magnitude than ex-post advice. However, when AI generated incorrect recommendations on initially right fills, ex-ante advice resulted in significantly smaller trust decrement than ex-post advice. Further differentiation of ex-post advice revealed an ordered pattern, where involved ex-post that actively disagreed in the decision process prompted the greatest trust adjustment magnitude, followed by ex-ante advice, with not-involved ex-post yielding the smallest magnitude. This ordering was consistent across the right-incorrect recognition pattern.

In addition, pharmacists’ trust dynamics demonstrated ecological validity of trust dynamics properties within a realistic verification context. The findings indicate that pharmacists’ trust in AI is dynamic and influenced by the timing, level of involvement, and situational performance of AI assistance during medication verification.

### Interpretation of Findings

#### Comparison Between AI Types

Comparison between the ex-ante and ex-post advice revealed a marginally higher magnitude for ex-ante advice compared to the ex-post advice, which aligns with the findings of Yin et al [27] that the ex-post advice resulted in superior diagnostic quality. When we further decomposed the ex-post advice paradigm into involved ex-post and not-involved ex-post to examine more nuanced trust dynamics associated with AI agreement and disagreement, the involved ex-post condition yielded the highest magnitude of trust adjustment, followed by the ex-ante advice, and then the not-involved ex-post condition. This pattern suggests that interruptive, disagreement-based interventions may exert a strong influence on trust. The ex-ante advice also required pharmacists to evaluate the AI’s accuracy during verification, leading to greater trust fluctuations than in the not-involved ex-post condition, which preserved workflow continuity and imposed no extra attentional demand.

#### Within Recognition Pattern Comparisons

We further analyzed trust adjustment behavior across AI recognition patterns, which considered both the correctness of the filled medication (*right fill* or *wrong fill*) and the accuracy of the AI’s prediction (*correct* or *incorrect*). Significant differences in trust adjustment across ex-ante and ex-post advice occurred when *the right fill was incorrectly rejected*. In this pattern, the ex-post advice led to a larger trust decrement compared to the ex-ante advice. For *the right fill was incorrectly rejected* pattern, significant differences in trust adjustment also occurred across interaction conditions. The involved ex-post condition led to the largest trust decrement compared to the other interaction conditions. The ex-ante advice showed a marginally greater trust decrement compared to the not-involved ex-post condition.

Understanding this pattern requires examining how each interaction condition responded in scenarios where AI incorrectly rejected the right drug. In the ex-ante advice condition, the AI’s incorrect prediction was visible from the start. Mismatch indicators (ie, red checkboxes) may have falsely anchored pharmacists’ attention toward an assumed mismatch and led to a moderate trust decrement [30,32]. In the involved ex-post condition, after pharmacists made the correct initial decision to accept the right drug, AI erroneously classified the mismatch and intervened with a false alert. This prompted the pharmacist to reconsider a correct initial decision, and the largest trust decrement was observed. This mirrors findings that incorrect ex-post alerts elicit strong trust decrements [42]. In contrast, the not-involved ex-post condition functioned as a passive agreement system. Under this condition, the AI silently agreed with the pharmacists’ incorrect initial rejection of the right drug. Here, the error was shared between pharmacists and the AI, and the smallest trust decrement was observed.

Operationally, an incorrect rejection of the right drug represents an unnecessary interruption in the dispensing process. Following the AI’s incorrect suggestion would require the pharmacist to reverify or refill a medication that was ready to be dispensed, introducing inefficiency. The larger trust decrement observed in this scenario likely does not stem from task consequence. When pharmacists’ accurate initial decisions were falsely contradicted by the involved ex-post condition, the error was salient, yielding strong trust decrements. Conversely, when both pharmacists and AI were wrong (not-involved ex-post), error attribution was shared, producing the smallest trust adjustment.

#### Validating Trust Dynamics Properties

Beyond evaluating AI interaction types, this study validated 3 trust dynamics properties—continuity, negativity bias, and stabilization [8,45,64]—with an ecologically realistic pharmacy verification environment. Prior validations have often relied on simplified tasks and nonexpert populations. By involving licensed pharmacists performing authentic pill verification, this study strengthens the ecological validity of trust-dynamics in human-AI interaction.

With licensed pharmacists, we demonstrated that trust evolves in a temporally dependent manner, such that trust at a given moment is shaped by prior interactions, strengthening following correct AI performance and declining after errors. We also observed negativity bias, reflecting the asymmetric influence of system performance, whereby trust is more sensitive to AI failures than to successes, making it difficult to build but easy to lose. Finally, pharmacists’ trust exhibited stabilization, showing a tendency to converge over repeated interactions as users developed more consistent expectations of the system’s behavior.

### Implications

These findings underscore the need for careful design of conditional intervention systems in safety-critical workflows. Ex-post advice assistance, implemented as a disagreement-triggered intervention, carries risks of confirmation bias, low revision rates, and negative reactions to incorrect alerts [40,43]. For pharmacists, who are expert decision makers and typically accurate, false AI interventions impose unnecessary cognitive costs. Frequent exposure to erroneous interventions may lead to over-skepticism toward the AI, undermining the intended safety benefit. Conversely, ex-ante advice avoids disruptive alerts but introduces anchoring and information-overload risks of Ex-Ante paradigms [30,31]. Both modalities present distinct trade-offs between autonomy support, cognitive load, and error-correction potential.

Designers of medication verification AI must consider these dynamics when determining when and how AI assistance should be delivered. For example, hybrid approaches, such as confidence-gated alerts, adaptive interventions, or selective suppression of low-confidence interruptions, may mitigate the negative impacts observed with the involved ex-post condition.

### Limitations and Future Directions

We acknowledge several limitations of this study and propose directions for future research. First, the AI system’s accuracy was experimentally reduced from its operational performance (98.5%) to 79% to ensure sufficient exposure to both correct and incorrect recommendations within a feasible number of trials. Although this approach enabled systematic examination of trust adjustments, it may limit generalizability to real-world settings where AI errors may be less frequent. In high-accuracy environments, errors may be more salient and unexpected, potentially eliciting different trust dynamics. Future research should investigate how trust evolves under near-realistic levels of AI reliability.

Second, in the involved ex-post condition, AI disagreement and workflow interruption were inherently coupled. Thus, the observed aggregated trust adjustment magnitude and trust decrement may reflect not only disagreement with the AI recommendation itself but also the cognitive and attentional disruption introduced by mid-task interventions. Future work should investigate the independent effects of disagreement content and workflow interruption on trust adjustment.

Third, the present analyses focused on self-reported trust adjustment, captured through trial-by-trial trust ratings, rather than subsequent behavioral adaptation to the AI support. As a result, the study does not explicitly highlight whether the trust pattern predicts behavioral responses, such as overriding AI recommendations, changes in decision strategies, or adjustments in reaction time. The asymmetric trust pattern observed in the study (negativity bias) may also relate to the broader literature on algorithm aversion, which suggests humans might respond differently to algorithmic mistakes, to an extent that they may disregard using systems even if they outperform humans [40,65]. Future work should investigate how moment-to-moment trust dynamics influence the subsequent decision-making behavior during repeated interactions with AI systems.

### Conclusions

This study examined pharmacists’ trust dynamics in response to variations in AI assistance timing and conditionality during the medication verification task. By comparing ex-ante advice and ex-post advice paradigms, and further decomposing ex-post advice into involved and not-involved conditions, the findings demonstrate that both timing and conditionality influence trust. In particular, ex-ante advice resulted in greater overall trust adjustment magnitude than in ex-post advice. However, when further decomposed, disagreement-based interventions that incorrectly challenge right expert decisions produced the largest trust decrements and greatest trust fluctuations.

These results extend prior work on AI advice timing by showing that the effectiveness of ex-post advice depends on the nature of AI involvement. While ex-post advice has been shown to enhance overall decision quality, the present findings indicate that the degree to which the AI agrees or disagrees with users’ initial decision plays a critical role in trust adjustments beyond the AI advice timing alone. For expert users performing safety-critical tasks, such as pharmacists performing medication verification, erroneous AI interruptions may influence trust more negatively than continuous AI support. This highlights the need to align AI intervention strategies with user expertise and task demands.

Finally, this work also validates the view that trust in AI is a dynamic, interaction-driven construct that evolves through repeated encounters with system behavior. By validating trust dynamics properties in an ecologically realistic pharmacy verification task, this study contributes to human-AI interaction research that emphasizes the importance of capturing moment-to-moment trust adjustments.

## Abbreviations

BNN: Bayesian neural network
CDSS: clinical decision support system
EMM: estimated marginal mean
HCI: human-computer interaction
NASA-TLX: NASA Task Load Index
NDC: National Drug Code
OFER: Occupational Fatigue Exhaustion/Recovery Scale
